# Physical therapists’ perspectives of patient values and their place in clinical practice: a qualitative study

**DOI:** 10.1016/j.bjpt.2023.100552

**Published:** 2023-10-05

**Authors:** Carla M. Bastemeijer, Johannes P. van Ewijk, Jan A. Hazelzet, Lennard P. Voogt

**Affiliations:** a Department of Public Health, Erasmus University Medical Center, Rotterdam, the Netherlands; b University of Humanistics, Utrecht, the Netherlands; c Department of Physical Therapy Studies, Rotterdam University of Applied Sciences, Rotterdam, the Netherlands

**Keywords:** Patient-centered care, Patient preference, Primary health care, Professional-patient relations, Qualitative research

## Abstract

**Background:**

In physical therapy practice patients and therapists exchange their perspectives on musculoskeletal health problems and their meaning for both of them. However, literature indicates that physical therapists find it difficult to enquire about the patients’ values during clinical encounters.

**Objectives:**

The aim of this study was to gain deeper insight into the perspectives of physical therapists about patient values.

**Design:**

Explorative qualitative focus group study.

**Method:**

Twenty-three physical therapists were interviewed in the Netherlands from March to May 2021. Two researchers analyzed the interviews and derived relevant codes. After an iterative process of comparing, analyzing, conceptualizing and discussing the codes, themes were identified through a thematic framework, illustrated with meaningful quotes.

**Results:**

Three major themes were identified: Humane, Tacit, and Responsive. It appeared that patient values play unconsciously a major role in daily practice and are associated with humanity, not technical or procedural aspects of the encounter. Responsive denotes that all values require interaction in which aligning with the individual patient forms the basis of treatment. Barriers for being responsive are identified as subthemes: Choices, Trust, Diverseness, and Boundaries.

**Conclusion:**

The concept of patient values appeared to be implicit. The professional intuitively attunes as a fellow human being to values and expectations of the individual patient. This study contributes to finding a balance and mutual reinforcement of implicit and explicit knowledge. With all found experiences and insights the concept of patient values became more explicit in physical therapy to create a framework for education and research in the future.

## Highlights

• Taking patient values into account is implicit and intuitive.
• Patient values are closely associated with humanity in care.
• Most barriers are experienced in being responsive.
• Guidelines seems to be at odds regarding uniqueness of patients.
• Systematic reflection on patient values is necessary in high quality care.

## Introduction

In physical therapy practice, patients and physical therapists exchange their perspectives on unique and personal musculoskeletal health problems. Whereas physical therapists often experience these problems primarily from an outsider stance, patients experience their health problems as an infringement on their individual lives.^1^, ^2^, ^3^

Good patient-professional interaction is a prerequisite for a deep understanding of patients with musculoskeletal pain and is positively related to better treatment outcomes, improved performance in daily living, and coping with pain.^4^^,^^5^ However, difficulties in patient-therapist interactions have been reported.^6^ Patients report healthcare providers do not seem to ‘care’ about their circumstances at times which makes them often feel neglected or not taken seriously.^5^^,^^7^, ^8^, ^9^, ^10^ Conversely, therapists discuss being confronted by patients’ beliefs, expectations, and attitudes as parts of patients’ individual values and conflicting professional knowledge and choices of themselves.^11^, ^12^, ^13^ In the clinical encounter both professional and personal values become part of an often implicit process of blending, sharing, colliding, or negotiating.^14^

A systematic review that focused on describing the content and meaning of patient values (PV) as part of patient-therapist interactions identified a preliminary taxonomy of PV.^15^ A qualitative study on the meaning of PV in physical therapy enriched this taxonomy by the categorization into eight elements; 1) uniqueness, 2) autonomy, 3) technically skilled professional, 4) conscientious professional, 5) compassionate professional, 6) responsive professional, 7) partnership, and 8) empowerment.^16^ Securing these values in patient-therapist encounters may further the quality of the professional interaction and improve patient-relevant outcomes. How PV and professional values become intertwined in physical therapy is largely unknown. Therefore, the following research questions were addressed:

- What is the meaning of PV for physical therapists in daily practice?
- How do physical therapists take PV into account in daily practice?
- What are barriers and facilitators for taking PV into account?

## Methods

### Design

This study was designed as a qualitative focus group study using Ritchie & Spencer's framework analysis.^17^ The 32-item COREQ (COnsolidated criteria for REporting Qualitative research) checklist was used to report the study.^18^

### Participants and setting

Convenience sampling via social media and telephone was used to recruit participants of primary care physical therapy practices across The Netherlands.^19^, ^20^, ^21^ Participants were eligible if they had more than 5 years working experience and no direct relationship with the researcher. The responding participants were asked to nominate candidates from their network/region who were contacted and asked to enroll into this study by the principal investigator (CB). The participants were informed about the aim and procedures of the study and provided verbal and written informed consent.

Ethical approval was given by the Institutional Review Board ErasmusMC Rotterdam, the Netherlands.

### Data collection and procedure

Two focus group interviews were scheduled, conducted, and recorded in March-May 2021 for each of the three groups of 7–8 participants (randomly composed). Interviews were held via an online videoconferencing tool because of Covid. The groups were moderated in Dutch by one of the researchers (LV, male/CB, female), both physical therapists for over 25 years, lecturers, PhD, and experienced and trained qualitative interviewers. Reflective notes were made during the interviews, such as common remarks, meaningful statements, and thoughts among the participants.

The first interviews were held for each of the three focus groups with the aim of collecting thoughts, experiences, and opinions regarding the concept of PV. The following topics were addressed;

- What is the meaning of PV for you in daily practice?
- How do you take PV into account in daily practice?
- What barriers and/or facilitators do you experience by taking PV into account?

Central themes of the first focus group interviews were identified^17^ by the researchers and then related and compared with the existing taxonomy to highlight differences and similarities.^16^ These outcomes were the starting point for the second focus group interviews.

The second focus group interviews were to gain more depth and detail into the discussion and were preceded by sharing the aggregated responses of the first interviews compared to the existing taxonomy.^16^ The groups could respond, complete, or reconsider their first answers. Subsequently, the elements of the taxonomy (uniqueness, autonomy, technically skilled professional, conscientious professional, compassionate professional, responsive professional, partnership, and empowerment) were explicitly discussed in depth. There were no repeat interviews. The thematic framework found as a result of data analysis was shared as a final member check with the participants for approval or adjustment.

### Data analysis

Framework analysis as described by Ritchie & Spencer was used to explore the acquired data, involving five interconnected stages; 1) familiarization, 2) identifying a thematic framework, 3) indexing, 4) charting, 5) mapping and interpretation.^17^ The units of analysis were the Dutch verbatim transcriptions of the video-recorded focus group interviews, which were transcribed using an automatic transcription tool, checked to ensure accuracy, and de-identified (CB). Two researchers (CB, LV) separately read the transcripts and reflective notes of the first interviews and coded meaningful words, sentences, or paragraphs. Individual interpretations were discussed and consensus was reached about understanding the research material. Major themes were marked, related to existing literature, and then discussed with the participants as a starting point for second interviews for more profound descriptions. Subsequently, all data were triangulated by analyzing (CB/LV), discussing, and relating the data to existing literature (CB/LV/JE/JH). The data were considered sufficiently rich and in-depth to draw conclusions (CB/LV/JE/LV). The third step was sorting the data on quotes and making comparisons within and between cases. The fourth stage, charting, involved lifting the quotes from their original context and arranging them under the major themes found. At the last stage, mapping and interpreting, the relationship was sought between the quotes and the data as a whole.

Trustworthiness of the study was addressed by enhancing credibility (member checking and triangulation), dependability (audit trail of procedures and processes), conformability (audit trail of data analysis), and transferability (thick descriptions).^22^

## Results

### Characteristics of participants

Twenty-seven physical therapists were eligible for participation of which 23 physical therapists of 21 Dutch primary care physical therapy practices participated based on their interest and their availability of time schedules. Participants were aged between 29 and 63 years (45 years on average), 11 being female and 12 being male with 6 to 41 years work experience (23 years on average) (Table 1).

**Table 1:** Characteristics of participants.

Participant	Interview FG1-6	Sex	Age (yrs)	Work (yrs) experience	Expertise
P1	1, 6	M	55	30	Physical Therapist/ Acupuncturist
P2	1, 5	M	59	38	Manual Therapist
P3	3, 5	F	43	21	Manual Therapist/ Oncology Manual Therapist
P4	2, 4	F	54	32	Manual Therapist/ Extended Scope Physical Therapist
P5	2, 5	M	47	26	Manual Therapist
P6	1, 4	F	34	11	Oncology Physical Therapist
P7	1, 5	F	54	33	Manual Therapist
P8	3, 6	F	32	12	Manual Therapist
P9	2, 6	M	29	6	Manual Therapist
P11	2, 5	F	36	14	Psychosomatic Physical Therapist
P12	3, 5	F	55	34	Manual Therapist/ Oncology/ Extended Scope Physical Therapist
P13	2, 6	M	40	16	Manual Therapist
P14	1, 5	M	35	12	Manual Therapist
P15	2, 6	F	32	11	Manual Therapist/ Extended Scope Physical Therapist
P17	3, 4	M	59	36	Manual Therapist
P18	3, 4	M	40	13	Physical Therapist
P19	1, 4	F	55	34	Pelvic Physical Therapist
P20	1, 4	M	49	27	Manual Therapist
P21	3, 4	M	33	13	Manual Therapist
P22	1, 5	F	43	21	Manual Therapist/ Extended Scope Physical Therapist
P24	2, 4	M	47	27	Sports Physical Therapist
P25	3, 6	F	63	41	Oncology Physical Therapist/ Lifestyle coach
P27	2, 6	M	31	10	Physical Therapist

Abbreviations: P, participant; FG, focus group; F, female; M, male; yrs, years.

**Fig. 1:**
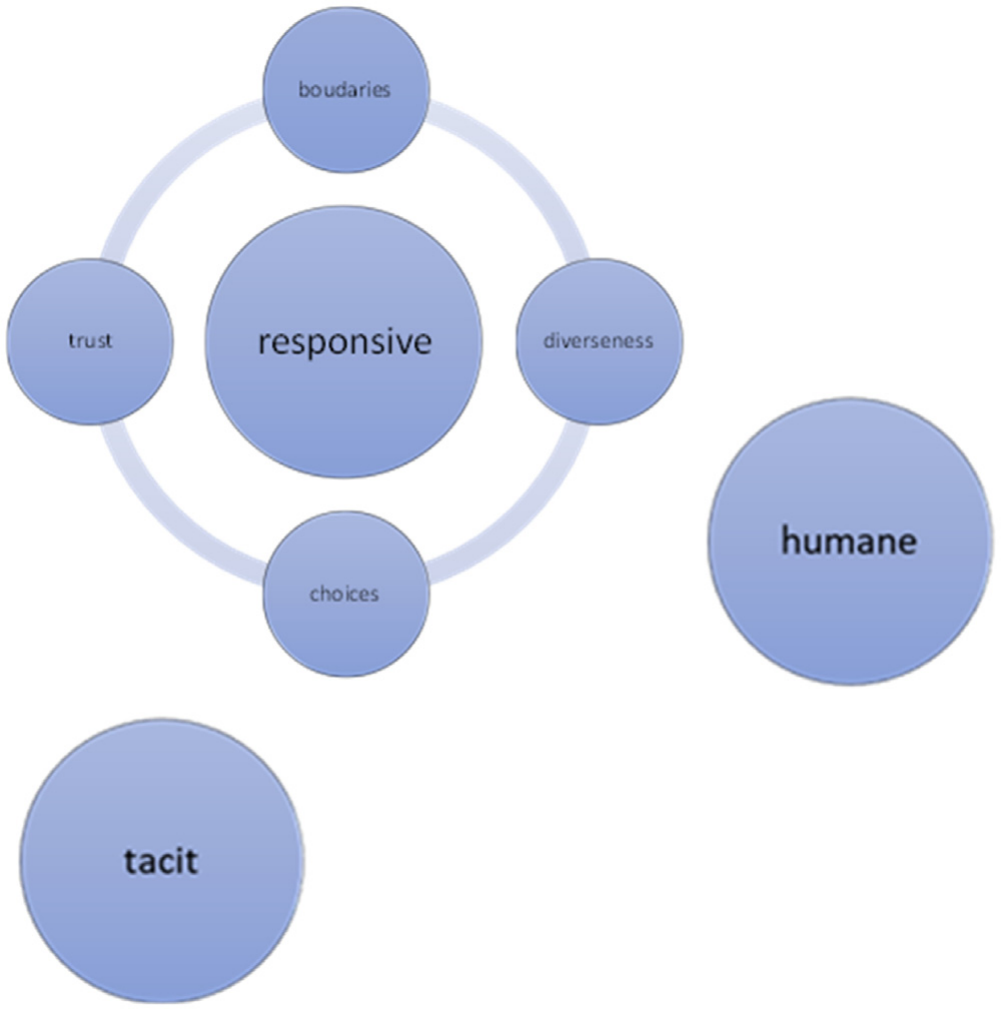
Thematic framework of patient values for physical therapists.

The interviews lasted 75–89 min (81 min on average). A thematic framework was identified as a result of the data. We found that the meaning of PV for physical therapists was mostly ‘Tacit’ and related to being ‘Humane’ and ‘Responsive’. Being responsive can be suppressed by barriers, categorized in four subthemes; a) trust, b) choice, c) diverseness, and d) boundaries (Fig. 1).

### Tacit

The interviews revealed the difficulty of defining the concept of PV with participants concluding that taking PV into account appears to be mainly implicit and intuitive.

P21 FG3: Of course, it is not an operationalized process…and that makes it so…fuzzy… but how is it secured? Yes, not as in a tangible thing… We secure it, I think… mainly unconsciously… in the relationship we build with our patients …

Apparently, participants act naturally or automatically on PV. According to the participants there is minor rationality or systematic reflection concerning this part of the encounter. Reckoning with PV is seen as a valuable skill which proceeds untaught and develops over the years and through experience.

### Humane

Participants believe that PV are important and closely associated with humanity within the consultation and much less with process aspects such as clinical reasoning, technical competency, or exercise therapy.

P18 FG3: … what the patient actually finds the most important, interaction between you and the patient … not just in the applied treatment. So I think, these values can be especially important in interaction, because patients feel you are kind and helpful and they feel comfortable with you …….. whether you are actually good at what you do or not… I tell my trainees, patients don't know that…

PV are seen by the participants in contrast to scientific evidence and the policy of health insurers which is associated with clinimetrics, guidelines, and protocols.

P24 FG2: I am not one for using questionnaires I have to say honestly…I think it is far more important to focus on the patient.

### Responsive

Being responsive appears to be an interconnective theme in the interviews. Participants express the importance of a committed and responsible execution of treatment and care including respecting uniqueness and autonomy. They feel responsibility for a fair and humane approach and dealing with boundaries such as idiomatic, cultural, or religious values; Physical therapists continuously attune to the patient.

P17 FG3: …the interaction between physical therapist and patient, that it will be driven by values of them both. They sometimes cross over, sometimes they even collide. For me it is the ability to finally say that there is something similar to fusion…. to achieve the best results for both. That means that the patient also has their input as well as me…

For an optimal patient-therapist relationship examples were given of continuous monitoring, adjustment, and assurance of PV and expectations with respect to physical therapists values and their professional boundaries. At the same time barriers are identified in that process and categorized into four subthemes; 1) choices, 2) boundaries, 3) diverseness, and 4) trust.

### Choices

At times, tensions arise in making choices or decisions in treatment. In reckoning with PV most barriers were experienced around this theme and the importance, but also the difficulty of shared decision making came to light here. For instance, when a patient has good experiences with hands-on therapy and a physical therapist is actually not open to that due to little scientific evidence, what choice will be made? Does the physical therapist stick to their point of view, in line with protocols and guidelines, with the risk of patient's feeling misunderstood due to different expectations? Or does the therapist go along with the values or experiences of the patient with the risk of inadequate or unnecessary treatment? The therapist experiences being trapped between the autonomy of the patient and the professional guidelines and protocols.

P7 FG1: …when you and the patient agree, starting a course of treatment, the patients has their own ideas, the things they need to do… and after two or three times… then you ask: How are you getting on? You hear; ‘I didn´t do those exercises because… I didn't have time for it’… The patient then moves into a passive role and puts the expectation onto me… which could be to the detriment of the whole trajectory…

### Boundaries

This theme is about perceived barriers in ending treatment, either on substantive or financial grounds, especially when the physical therapist appoints (moral) professional boundaries that patients would not take into consideration. Examples of participants include stopping treatment in the event of insufficient progress, or too many psychosocial problems as a hindrance factor, or because the final goal has been achieved despite available financial remuneration. Or conversely, when a goal cannot be achieved actually due to unavailable remuneration.

P20 FG1: … the patient simply felt that they were entitled to the total number of treatments in their insurance package (regardless of whether they were necessary or not).

P19 FG1: …the defining aspect, how someone is insured… Some people have such limited insurance policies and there is so little coverage in a basic insurance policy that you already have a conflict of conscience…

Being genuinely interested and putting the patient foremost can also cause problems according to the participants. When physical therapists become too compassionate it is more difficult to be totally honest, to confront the dilemma and refuse to treat more often than is actually necessary.

P24 FG2: … I listen completely to the patient's story and their social situation. Maybe I have carried on treating them for too long… in my eyes….or maybe I haven't been clear enough about my competence, my professional boundaries…actually being too sympathetic…

### Diverseness

Participants experience barriers regarding uniqueness of patients, their values, and the complexity of health problems on the one hand, and professional guidelines and protocols on the other hand. The use of guidelines seems to be at odds with the elements of responsiveness and uniqueness of the taxonomy.

P18 FG4: Non-specific low back pain for example… it is clearly described what should be done in these cases…Well, I think that cases can be so complex that you can throw protocols out of the window, so to speak … because patients, they are unique, that it is precisely why it is actually different for each individual.

Furthermore, the time required to fully discuss and align treatment with a patient is often limited, even more when the physical therapist experiences barriers in language, religion, or culture.

P20 FG1: … the whole spectrum of PV… from A to Z … and you try to meet all of them within half an hour, … Mmm, then I actually feel that I have to make concessions, often in the depth of their story. And when this happens… did I really deliver the quality in care that I endeavor to achieve?

Also, some patients have a lack of self-reliance by nature or insufficient health literacy. To empower these patients, they may need to be treated for a little longer or social networks need to be involved. Only time or opportunity is often unavailable.

P4 FG2: … if the patient has limited health literacy and finds it better to be taken by the hand, so to speak. While you would prefer, of course, that the patient could have a better understanding of their complaints themself. If they don't have the necessary competencies… what should you do? I find that rather difficult…

### Trust

A lack of confidence in professionalism is seen as a barrier for patient-therapist relationships, the participants highlighted a number of reasons; 1) based on age or little work experience of the physical therapist, 2) due to the position, for example as junior therapist, and 3) if the referrer has already determined through the referral letter or during the consultation which treatment should take place. Conjointly, the patient may come in with prior information or own ideas and is no longer open to the professional's perspective and knowledge.

P7 FG1: Now, I see a patient in front of me, who has a rock solid belief that she has fractured something in her neck; she has already been on a number of multidisciplinary trajectories …if she refuses to budge then we cannot move forward.

In addition, participants sometimes experience regulations or advice from their profession as a lack of confidence in their clinical decision-making. Examples of being cautious with high cervical spine manipulation or hands-on treatment in general are mentioned. It feels like an infringement of professional autonomy and self-confidence.

P11 FG2: In physical therapy education… massage, hands-on treatment, well, you could say, it's almost not done anymore. … A GP said to me: What a pity, you could improve people's attitude to their health just by making contact, by putting your hands on their back.

### Taxonomy

In addition to the themes mentioned above, the data of the first focus group interviews demonstrate a representation of all main themes found in earlier research of PV in physical therapy (uniqueness, autonomy, technically skilled professional, conscientious professional, compassionate professional, responsive professional, partnership, and empowerment). Sometimes explicit but more often implicit. The second round of interviews gave more depth and detail into the discussion and was preceded by sharing the aggregated responses of the first interviews, explicitly compared to the existing taxonomy. All participants recognized and confirmed the unique themes and the taxonomy as a whole. The interwovenness of elements was illustrated by clinical examples.

P22 FG1: …and then I start a physical examination. And when we have a connection, if that is the case, I will explain a little. By this I actually help them to understand, that there is some recognition and navigation, so that they can think with me. Well, then we can discuss how we are actually going to handle the medical care and then I will give an approximate time frame, how long it will require before they notice any progress… and after two or three consultations we can evaluate whether progress has been made or whether a change of course should be made.

## Discussion

Understanding how PV and professional values become intertwined is essential for physical therapy practice. PV are fundamental characteristics of concepts like ‘evidence based care’, ‘value based care’, and ‘patient centered care’ and should be acknowledged in clinical care.^3^^,^^23^^,^^24^

### What is the meaning of PV for physical therapists in daily practice?

The analyzed data show that PV correspond to the previously found, in physical therapy enriched taxonomy^16^ and that patients must been seen from a biopsychosocial stance.^25^^,^^26^ Responsive is, as in previous studies, recognized as an interconnective element. All values require an interaction where alignment with the individual patient forms the basis of the treatment.^27^ The data confirm that Responsive includes elements such as culture, language, religion, and level of education^15^ and the requirement of ongoing aligning and assessment for care matching the patient goals.^28^ A good therapist interaction, as an expression of partnership and human interaction, will contribute to positive health outcomes.^4^^,^^5^^,^^29^

### How do physical therapists take PV into account in daily practice?

That values are often preverbal and are implicitly taken into account aligns in the phenomenon of tacit knowledge.^30^ This phenomenon often contains (cultural) values, experiences, and attitudes and become visible in actions, intuition, and routines. Explicit knowledge distinguishes itself from tacit knowledge by its objectivity through literature and science. Tacit knowledge is not by definition sufficient, it requires development, learning, reflection, and correction.^31^ Participants agree that taking PV into account develops by work and life experience, which corresponds with the development of tacit knowledge.^30^

### What are barriers and facilitators for taking PV into account?

This study confirms that patient alignment can be both a facilitator and a barrier in patient-professional interaction.^29^ Noticeably, the description of PV by practical examples often show the tension between taking PV into account and safeguarding professional values. This often leads to uncomfortable situations, difficult conversations, or even discontent. The integration of different kinds of ‘knowledge’ (scientific evidence vs. moral values) do not easily merge and sometimes lead to clinical dilemmas.^11^^,^^12^^,^^32^^,^^33^ This refers to literature about tinkering. Good care is always a matter of tinkering with different, sometimes competing goods which calls for momentary judgments across changing situations.^34^^,^^35^ The importance, but also the difficulty of shared decision making comes to light in the results.^36^^,^^37^

### Methodological considerations

To our knowledge, this is the first study aiming to gain more insight into the perspectives of physical therapists about PV which were determined in previous qualitative and literature research.^16^ The study contributed to the consideration of PV from three perspectives; literature, patients, and healthcare providers.

Weaknesses of this study involve the choice of including participants by convenience sampling via social media and telephone. This could have given an incomplete representation of results by the fact that perhaps these participants had read about or were more interested in the subject than on average. Furthermore, physical therapy is practiced in a wide variety of contexts. The findings of this study in primary care may not translate to other contexts, for example different health systems or different cultural settings.

### Implications for education and clinical practice

Becoming a responsive physical therapist may require specific educational strategies and training. These ‘soft’ skills are less tangible and consists of multiple components such as attitude, communication, and etiquette which need to be addressed accordingly. It is important to share the gained explicit knowledge about PV in education to make actions more transparent and transferable.^38^^,^^39^ Additionally, further research is needed in how to find a more equal balance between explicit scientific knowledge and implicit tacit knowledge. The professional experiences a conflict between these two worlds on a daily basis and feels mainly valued on the objectifiable, scientific basis of the profession. By considering the patient's complaint, pain, and situation, and explaining the professional's viewpoint, a feeling of trust and co-creation ought to be sought; a collaboration in which both patient and physical therapist influence the process ending with a valuable result.^6^^,^^28^

## Conclusions

The findings of this study help physical therapists to understand what PV mean in physical therapy and how they can take them into account. Tacit, Humane, and Responsive are the identified main themes. PV are tacit knowledge; the competent professional aligns continuously, intuitively, as a fellow human being to the values and expectations of the individual patient to achieve optimal care and treatment. Within Responsive barriers are identified, categorized into four subthemes; 1) choices, 2) boundaries, 3) diverseness, and 4) trust. Until now, current scientific insights attach great value to explicit knowledge. Although explicit knowledge and skills may be very objective and transferable, there is a risk of ending in rigid regulations and overlooking essential elements in the therapeutic process. This research provides a better insight into the important role for PV, professional reflection and the presence of tacit knowledge. The study intended to help us find a balance and mutually reinforce implicit and explicit knowledge. With all the experiences and insights mentioned, the concept of PV in physical therapy is given more substance to create a framework for education and research.

## Conflicts of interest

The authors have no conflict of interests that could have influenced this paper.
